# Paperclip RNAs bypass clathrin mediated endocytosis in insect and fungal cells

**DOI:** 10.1371/journal.pone.0359888

**Published:** 2026-10-28

**Authors:** Nazanin Amanat, Bliss M. Kernodle, Brigo Castillo, Alison Tayler, Mark Belmonte, Steve Whyard

**Affiliations:** 1 Department of Biological Sciences, University of Manitoba, Winnipeg, Manitoba, Canada; 2 Joint Genome Institute, Lawrence Berkeley National Laboratory, Berkeley, California, United States of America; Guangzhou University, CHINA

## Abstract

Insects and fungi are among the most destructive pests and pathogens affecting agriculture and public health. Although chemical pesticides have long been the primary means of control, their broad‑spectrum activity and widespread use have accelerated resistance in many systems. RNA interference (RNAi) has therefore emerged as a promising species‑specific alternative. RNAi is initiated by double‑stranded RNA (dsRNA), which triggers sequence‑directed degradation of target transcripts. However, RNAi efficiency varies markedly across species, influenced by nuclease activity, dsRNA length, and the cellular uptake route. We previously showed that long dsRNA uptake in *Aedes aegypti* depends on clathrin‑mediated endocytosis (CME), whereas paperclip RNA (pcRNA), a compact dsRNA structure developed in our laboratory, bypasses CME and enters cells through a distinct pathway. Here, we compared the uptake and RNAi performance of two dsRNA formats, long dsRNA (210–230 bp) and pcRNA (21–23 bp), in three representative species: *Aedes aegypti* (mosquito), *Spodoptera frugiperda* (moth), and *Sclerotinia sclerotiorum* (fungus). To assess cellular uptake of these dsRNAs, we used two CME inhibitors, chlorpromazine (CPZ) and bafilomycin A1 (BafA1), along with fluorescein‑labeled dsRNAs. CME inhibition prevented long dsRNA from entering the cytoplasm and abolished its ability to induce RNAi. In contrast, pcRNAs were readily internalized and produced significant gene knockdowns under both inhibitor treatments. Microscopy further revealed that all three cell types, pretreated with either CPZ or BafA1, prevented long dsRNA from entering the cytoplasm. pcRNA, consistently reached the cytoplasm and showed superior uptake across all species. Together, these findings demonstrate that pcRNA uses an uptake mechanism distinct from long dsRNA, enabling it to circumvent CME-dependent barriers. This unique internalization route likely reflects its compact size and structural configuration and highlights pcRNA as a promising platform for improving RNAi-based pest and pathogen control.

## Introduction

Insect pests and fungal pathogens are two of the most significant threats to global agriculture, accounting for approximately 20% [1] and 14% [2] of annual crop yield losses, respectively. These pressures are expected to intensify as agricultural systems strive to meet the demands of a growing global population under increasing climate variability. Currently, chemical pesticides remain the primary means of managing both insect and fungal threats. However, broad-spectrum insecticides, including neonicotinoids, organophosphates, carbamates, and pyrethroids, can harm non-target organisms, reduce biodiversity, and degrade soil health [3–5]. Neonicotinoids, for example, can directly kill honeybees, and even sublethal exposures impair colony function by increasing worker mortality, weakening social immunity, and contributing to elevated queen loss [6–8]. Importantly, concerns extend beyond ecological impacts: many widely used insecticide classes are associated with adverse human‑health outcomes due to their neurotoxicity, environmental persistence, and potential for chronic exposure [9,10].

Broad‑spectrum fungicides frequently enter aquatic environments through spray drift, drainage, and surface runoff [11], where they can disrupt non‑target organisms because many of these compounds act on highly conserved cellular pathways [12]. Strobilurin residues, for example, have been shown to impair survival and development in larval Great Plains toads (*Anaxyrus cognatus*) [13–15], and similar adverse effects have been reported for other amphibians and aquatic invertebrates exposed to broad‑spectrum fungicides such as chlorothalonil [16]. These concerns extend to terrestrial systems as well. Field‑relevant concentrations of several fungicides can cause sublethal behavioural and physiological effects in bumble bees [17], raising questions about pollinator health in agricultural landscapes. Some fungicide classes also pose risks to mammals, including humans: succinate dehydrogenase inhibitor fungicides have been linked to mitochondrial dysfunction and tumor formation in both people and dogs [18–23].

Beyond these off-target effects, the continued reliance on conventional pesticides has accelerated the development of resistance across numerous insect orders [18–20,24,25] and plant pathogenic fungi [26–29]. As resistance increases, growers often respond by applying chemicals more frequently or at higher doses, a strategy that can raise production costs and exacerbate environmental harm. Collectively, these challenges highlight the urgent need for alternative crop protection strategies that effectively manage pests and pathogens while safeguarding beneficial species and ecosystem health.

One promising alternative is RNA interference (RNAi), a highly conserved post-transcriptional gene silencing mechanism. Initially developed as a reverse-genetics tool in *Caenorhabditis elegans*, RNAi has since been adapted for diverse applications, including antiviral therapeutics and oncology treatments [30–32]. Its inherent sequence specificity has also positioned RNAi as an attractive platform for next-generation crop protection. Unlike traditional chemistries, RNAi-based biopesticides selectively silence essential pest genes, enabling effective control with minimal off-target effects [33–35].

A practical approach for deploying RNAi in agronomic settings is spray-induced gene silencing (SIGS) [36]. In SIGS, double-stranded RNAs (dsRNAs) with sequence homology to essential pest genes are topically applied to plant surfaces, aligning with existing agronomic practices. Upon uptake by the pest, long dsRNA is processed by Dicer into 21–23 bp small interfering RNAs (siRNAs), which are incorporated into the RNA-induced silencing complex (RISC). The Argonaute protein then guides the cleavage of complementary mRNA, suppressing target gene expression [37,38].

SIGS has demonstrated strong efficacy against major fungal pathogens such as white mold (*Sclerotinia sclerotiorum*), grey mold (*Botrytis cinerea*), rice blast (*Magnaporthe oryzae*), Asian soybean rust (*Phakopsora pachyrhizi*), and Fusarium head blight (*Fusarium graminearum*) [39–42]. Similarly, insecticidal activity has also been demonstrated against the diamondback moth (*Plutella xylostella*) and the Colorado potato beetle (*Leptinotarsa decemlineata*) [43], with the latter leading to the approval of the first foliar dsRNA biopesticide, Calantha [44]. Despite this cross-kingdom potential, RNAi efficiency varies widely among species, limiting its reliability as a universal pest control strategy [45].

For example, coleopteran insects are among the most RNAi-sensitive insects, with *L. decemlineata* consistently exhibiting high mortality following SIGS treatment [45]. In contrast, lepidopterans are largely refractory to RNAi due to high midgut nuclease activity and inefficient dsRNA uptake [46]. Similar variation occurs in fungi: *S. sclerotiorum*, *B. cinerea,* and *M. oryzae* readily take up and process exogenous dsRNAs [39–41], whereas *Zymoseptoria tritici*, a major wheat pathogen, shows no uptake despite possessing the necessary RNAi machinery [47]. Other fungal species with minimal dsRNA uptake include *Trichoderma virens* and *Colletotrichum gloeosporioides* [48]. These disparities highlight the need for improved dsRNA structures or delivery systems that enhance cellular internalization.

To address this challenge, we employed a novel dsRNA structure termed paperclip RNA (pcRNA), characterized by a short, linear double-stranded region (23–25 bp) flanked by looped ends [49]. This looped‑end architecture folds into a compact, hairpin‑like structure that differs markedly from conventional linear dsRNAs. In this study, we demonstrate that pcRNAs effectively silence target genes in cultured cells of a lepidopteran crop pest (moth), a dipteran disease vector (mosquito), and a plant-pathogenic fungus (*S. sclerotiorum*) under conditions where conventional long dsRNAs fail. We further characterize the intracellular localization of pcRNAs by tracking their uptake and trafficking through confocal microscopy. Together, these findings reveal robust cross-kingdom activity in economically significant pests and highlight the potential of pcRNA to overcome RNAi recalcitrance in agricultural and public health applications.

## Materials and methods

### Cell and fungal culturing

CCL125 cells (ATCC), an epithelial cell line derived from *Aedes aegypti* (Yellow fever mosquito) larvae, were maintained in Minimal Essential Medium (MEM/EBSS; Cytiva) supplemented with 20% (v/v) heat-inactivated fetal bovine serum (HI-FBS; Cytiva). Cultures were grown in T-75 flasks at 28°C in 5% CO_2_ and 80% relative humidity. Growth medium was refreshed twice weekly, and cells were passaged at a 1:3 ratio once per week following ATCC guidelines.

Sf9 cells (ATCC), an epithelial cell line derived from ovarian tissue of *Spodoptera frugiperda* (Fall Armyworm) pupae, were maintained in Grace’s Supplemented Insect Medium (Gibco) supplemented with 10% (v/v) heat-inactivated fetal bovine serum (HI-FBS; Cytiva). Cultures were grown in T-75 flasks at 28°C, without CO_2_ and 80% relative humidity. Growth medium was refreshed every 2–3 days, and cells were passaged at a 1:5 ratio according to ATCC protocols.

*Sclerotinia sclerotiorum* cultures were obtained from the Morden Research and Development Centre (Agriculture and Agri‑Food Canada). Ascospore-bearing apothecia were produced by carpogenic germination of sclerotia on a nutrient-rich germination medium and incubated on wet sand at 20°C. Following isolation, ascospores were stored at 4°C until use. For culture initiation, ascospores (1 × 10^5^ mL^-1^) were plated onto ¼ strength potato dextrose agar (PDA) and allowed to germinate. After 3 days of growth, actively expanding mycelial fronts were sampled by collecting a 1 mm^2^ plug using a pipette tip for use *in-vitro* liquid culture growth experiments as described below.

### Design and synthesis of insect and fungal dsRNA constructs

Total RNA was extracted from insect cells (CCL125 and Sf9) cells using the GeneJET RNA Purification Kit (Thermo), and cDNA was synthesized with qScript cDNA SuperMix (Quanta). Total RNA was extracted from *S. sclerotiorum* using the RNeasy Plant Extraction Kit (Qiagen) and treated with RNase-Free DNase (Qiagen) according to the manufacturer’s instructions. cDNA synthesis was performed using the qScript cDNA Synthesis Kit (Quantabio).

Long dsRNA templates for *in vitro* transcription were generated by PCR amplification of ~230 bp gene fragments from the cDNA using gene-specific primers containing T7 promoter sequences. Target genes were selected based on prior observations indicating that the corresponding dsRNA doses produced approximately 50% transcript knockdown, enabling detection of transcript-level perturbations in the inhibition assays described below (targets: *A. aegypti* – *Snf7* homolog (NCBI accession XM_021841452.1); [49] *S. frugiperda* – *PDZ domain-containing protein GIPC3-like* (hereafter referred to as *PDZ*; NCBI accession XM_035573157.2); *Sclerotinia AB-HYDROLASE 3* (SS1G_01703, XM_001597459.1).

pcRNAs were designed following Abbasi *et al*. [49], consisting of 23 bp complementary regions flanked by 9-nt loops to promote intramolecular folding into the pcRNA structure. To generate the pcRNA and siRNA templates, ultramer oligonucleotides corresponding to the two complementary strands were designed, synthesized (Integrated DNA Technologies), and annealed to produce double-stranded transcription templates. The pcRNA and siRNA sequences were chosen from within the long dsRNA region and optimized using IDT’s custom Dicer-substrate siRNA design tool.

*In-vitro* transcription was performed using the MEGAscript RNAi Kit (Ambion) according to the manufacturer’s instructions. Long dsRNAs were purified using the kit’s spin columns, whereas pcRNAs and siRNAs, being too short to efficiently bind the spin columns, were purified by ethanol/sodium acetate precipitation [50]. All dsRNA formats (long dsRNA, siRNA, pcRNA) were verified by gel electrophoresis to be pure and of the expected size prior to use.

### Fluorescent labeling of long dsRNA and pcRNA

Fluorescently labeled long dsRNA and pcRNAs were synthesized using the Fluorescein RNA Labeling Mix (Roche). To increase labeling efficiency, the incubation period with the fluorescein master mix was extended to 18 h (overnight), rather than the standard 4 h recommended by the manufacturer. Labeled RNAs were purified by ethanol/sodium acetate precipitation [50].

### RNAi knockdown assays in insect cells

To compare the RNAi efficacy of different dsRNA structures, long dsRNA (~220 bp), pcRNA (23 bp), and siRNA (21 bp) constructs targeting the *Snf7* or *PDZ* genes were tested in their respective insect cell lines. Cells were seeded into six-well plates and allowed to adhere for 24 h, then washed with 2 mL of 1x PBS. For positive controls, cells were transfected using Lipofectamine® RNAiMAX and Opti-MEM media (Invitrogen) to ensure the effective uptake of dsRNAs construct. 20 pmol for long dsRNA and 200 pmol for pcRNA and siRNA, separately were added to Opti-MEM media to a final volume of 150 μl per well. In a separate microcentrifuge tube, 9 μl of Lipofectamine® was added to 141 μl of Opti-MEM media per well, mixed gently, and incubated at room temperature for 5 minutes. Then, the two mixtures were combined, mixed gently, and incubated at room temperature for 5 minutes. 250 µL of the mixture was administered to each well. For the rest of the treatment, cells were treated with 20 pmol long dsRNA, 200 pmol siRNA, or 200 pmol pcRNA. The ten-fold higher molar concentration of pcRNA and siRNA approximated the number of siRNA molecules generated intracellularly from long dsRNA processing. After 48 h, cells were harvested for qRT-PCR analysis using gene-specific primers (described below). Negative controls consisted of long dsRNA, pcRNA, or siRNA targeting the *GUS* gene, an *E. coli* gene with no sequence identity to insect genomes.

### Uptake pathway inhibition assays in insect cells

To investigate the cellular uptake mechanisms of the differently structured dsRNAs, Sf9 and CCL125 cells were pre-treated with inhibitors of clathrin- and caveolin-mediated endocytosis. Chlorpromazine (CPZ, 10 µM), bafilomycin A1 (BafA1, 0.2 µM), and methyl-β-cyclodextrin (MβCD, 1 mM) was applied for 30 min at 28°C, and cells were then exposed to long dsRNA or pcRNA under the conditions described above. After 48 h, cells were harvested and transcript knockdown was quantified by qRT-PCR, described below. Positive controls received target dsRNA without inhibitors, while negative controls received dsGUS. To ensure that the concentrations of CPZ, BafA1, and MβCD used in all cell‑based assays were non‑cytotoxic, viability testing was performed using the MTT Assay Kit (Abcam) according to the manufacturer’s instructions. All inhibitor doses selected for RNAi and imaging experiments were confirmed to maintain cell viability comparable to untreated controls.

### Uptake pathway inhibition assays in *Sclerotinia sclerotiorum*

Fungal plugs (1 mm^2^) were excised from the actively growing mycelial fronts of 3-day-old *S. sclerotiorum* cultures growing on ¼ strength potato dextrose agar. Each plug was transferred to a 15mL tube containing 3 mL of ¼ strength potato dextrose broth supplemented with 50 µg/mL ampicillin. Cultures were treated with CPZ (10 µM), BafA1 (0.2 µM), or MβCD (2 mM) and incubated for 2h. Long dsRNA or pcRNA targeting Ss-ABH3 (500 ng/mL) was then added. Negative controls received dsRNA targeting GFP (Green Fluorescent Protein from *Aequorea victoria*) or GUS. Tubes were wrapped in aluminum foil and incubated at 17°C for 72 hours on a rocking platform.

### RNA extraction and cDNA synthesis for qRT-PCR analyses

After 72 h, fungal cultures were rinsed thoroughly with ddH_2_O to remove media and immediately flash frozen in liquid nitrogen. Total RNA was extracted using the RNeasy Plant Mini Kit (Qiagen). Genomic DNA contamination was removed using TURBO™ DNase (Thermo Scientific). Genomic DNA contamination was assessed by PCR using untreated RNA as the template: amplification indicated residual DNA. After DNase treatment, no PCR product was detected, whereas cDNA synthesized from the DNase‑treated RNA again produced the expected amplicon, confirming complete DNA removal. Total RNA from insect cells was extracted using the GeneJET RNA Purification Kit (Thermo Scientific) and genomic DNA contamination was removed and verified as described above. cDNA was synthesized using the qScript cDNA synthesis kit (Quantabio). qRT‑PCR analyses were then conducted using SsoFast EvaGreen Supermix (Bio‑Rad) on a Bio‑Rad CFX Connect or CFX96 Real‑Time PCR System. cDNA was diluted 1:4 prior to amplification. For *S. sclerotiorum*, reactions were run under the following conditions: 95 °C for 2 min; 40 cycles of 95 °C for 5 s, 55 °C for 5 s, 72 °C for 5 s; final extension at 72 °C for 3 min. Melt‑curve analysis (65–95 °C, 0.5 °C increments) confirmed specificity. Relative transcript abundance was calculated using the ΔΔCq method with *Sac7* (SS1G_12350, XM_001587270.1) as the reference gene. Primer sequences are provided in S1 Table.

For insect cells, qRT‑PCR was performed using the following cycling conditions: 95 °C for 3 min; 40 cycles of 95 °C for 10 s, 55 °C for 30 s. Melt‑curve analysis (55–95 °C, 0.5 °C increments) verified specificity. Transcript levels of *PDZ* and *Snf7* were quantified using the ΔΔCt method using *28S rRNA* as the internal reference. All values were normalized to the average transcript abundance of untreated cells, or to cells treated with vehicle-only for the inhibitor assays. Each treatment included six biological replicates, with triplicate technical reactions. Primer sequences are listed in S2 Table.

### Confocal localization of fluorescein-labeled dsRNAs

To visualize intracellular localization of dsRNA in the presence of uptake inhibitors, insect cells were seeded onto coverslips 24 h prior to treatment. After washing with 2 mL of 1x PBS, cells were incubated with uptake inhibitors as described above, then exposed to fluorescein-labeled long dsRNA or pcRNA targeting *Snf7* in mosquito cells, or *PDZ* in moth cells. Cells were imaged 20 min after incubation with dsRNAs.

For fungal localization assays, ascospores (10 µL of 10^6^ ascospore/mL) were applied to 12-well culture plates containing 400 µL ¼ strength PDA. Uptake inhibitors were added 14 h after germination, and 2 h later, fluorescein-labeled long dsRNA or pcRNA was applied. Hyphae were imaged 20 min after RNA addition.

Imaging for both insect cells and fungal hyphae was performed using a Zeiss LSM 700 confocal laser scanning microscope equipped with a Zeiss Imager M2 at 60x magnification, using 488 nm excitation and 516 nm emission detection. Z-stack images were acquired and analyzed to accurately resolve the signal localization on or within the cells.

### Cytoplasmic (non-endosomal) fluorescence quantification

Fluorescein intensity was measured from maximum‑intensity projections of Z‑stack images acquired on a Zeiss LSM 700 confocal microscope (Imager M2). All analyses were performed in ImageJ/Fiji (version 1.54t). For each cell, the fluorescein channel was isolated and the following measurements obtained: 1) Total fluorescence (TF): A whole‑cell ROI was drawn to encompass the entire cell, and TF recorded as the integrated density (sum of pixel intensities) within that ROI. 2) Background fluorescence (BF): A cell‑free region in the same image was used to obtain mean background intensity. BF for each cell was calculated as mean background intensity × ROI area.


$$\begin{array}{c} Corrected\text{\textasciitilde{}}total\text{\textasciitilde{}}fluorescence=TF-BF \end{array}$$

Endosomal fluorescence (EF): When punctate endosomal signal was present, endosomes were identified using a consistent threshold across all images to generate endosomal ROIs. EF was measured as the integrated density of all endosomal ROIs within the cell.


$$\begin{array}{c} Cytoplasmic\text{\textasciitilde{}}fluorescence\;=\;Corrected\text{\textasciitilde{}}TF-EF \end{array}$$

If no endosomes were detected, EF was set to zero, and cytoplasmic fluorescence equaled the corrected TF.

## Results

### RNAi activity of long dsRNA and pcRNA structures

The RNAi efficiencies of long linear dsRNA and pcRNA were evaluated in three cell types: CCL125 cells from *Aedes aegypti*, Sf9 cells from *Spodoptera frugiperda*, and fungal hyphae of *Sclerotinia sclerotiorum*. Knockdown was measured by quantifying transcript levels of *Snf7* in CCL125 cells, *PDZ* in Sf9 cells, and *AB-HYDROLASE 3* in *Sclerotinia*, each normalized to *28S rRNA* for insect cells and *Sac7* for *Sclerotinia* (Fig 1). These gene targets were selected because they represent validated or strongly supported candidates for pesticidal RNAi applications. The *snf7* gene has been demonstrated to be an effective RNAi target for corn rootworm control, providing protection *in planta* when targeted by dsRNA [51]. Both *Snf7* and *PDZ* encode proteins involved in intracellular trafficking, and genes associated with this cellular process have been identified by Velez et al. (2018) [52] as particularly effective RNAi targets in insects. *PDZ* was therefore selected as a potential target that may similarly prove effective in insects, although this has not yet been experimentally confirmed. For *Sclerotinia*, AB‑HYDROLASE 3 was chosen because dsRNA targeting this gene has previously been shown to suppress fungal growth in canola [39]. In addition to their potential or proven pesticidal relevance, all three genes have consistently produced moderate but reproducible knockdown in our prior cell‑based assays, making them suitable markers for detecting perturbations in RNA‑mediated silencing [39,49]. Different target genes were selected for each species because comparable knockdown levels could not be achieved when targeting orthologous genes in the two insect cell lines, and the preferred fungal target lacked clear orthologues in insects. Accordingly, *Snf7*, *PDZ*, and *AB-HYDROLASE 3* were used as species‑appropriate markers of RNAi efficacy. In CCL125 cells, long dsRNA and pcRNA reduced *Snf7* transcript levels by 53% and 63%, respectively, with no significant difference between the two RNA structures (Fig 1A). In Sf9 cells, long dsRNA and pcRNA decreased *PDZ* transcript abundance by 42% and 60%, and this difference was likewise not statistically significant (Fig 1B). In both insect cell lines, siRNAs containing the same 21‑bp sequence did not produce significant knockdown (S1 Fig A), unless they were delivered directly to the cells using Lipofectamine-mediated transfection (S1 Fig B).

**Fig 1:**
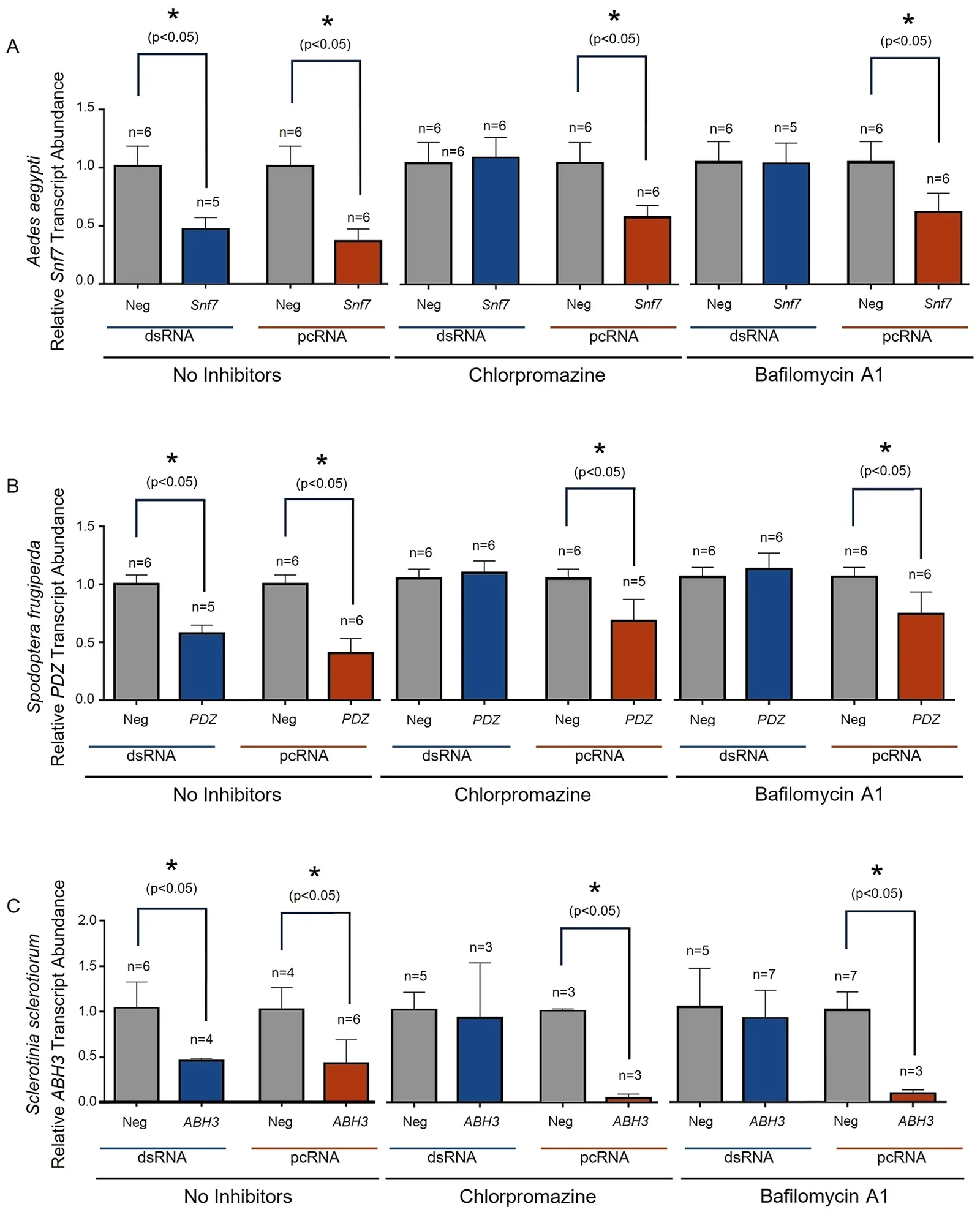
Transcript knockdown by long dsRNA and pcRNA in cultured *Aedes aegypti* (CCL125), *Spodoptera frugiperda* (Sf9) cells, and *Sclerotinia sclerotiorum* hyphae, both without inhibitors and following chemical inhibition of clathrin-mediated endocytosis. (A) *Snf7* transcript levels in *A. aegypti*. (B) *PDZ* transcript levels in *S. frugiperda*. (C) *AB-HYDROLASE 3* transcript levels in *S. sclerotiorum*. For the inhibitor assays, cells or hyphae were pre-treated with chlorpromazine or bafilomycin A1. Transcript abundance was quantified by qRT-PCR and normalized to *28S rRNA* (insects) or *Sac7* (fungus) and expressed relative to untreated controls. Negative control (Neg) treatments consisted of cells or hyphae exposed to non-specific (GUS) long or pcRNA, with vehicle-only treatments serving as the controls for inhibitor assays. Values represent the means ± s.e.m; n denotes biological replicates per treatment, each consisting of 3 technical replicates. Asterisks indicate both statistically significant differences (one-way ANOVA, Tukey HSD, p < 0.05) and a large size effect (Hedges’ g > 2.0).

In *Sclerotinia*, treatment with long dsRNA or pcRNA resulted in similar transcript reductions of approximately 55%, with no significant difference between the two structures (Fig 1C). siRNA targeting the same sequence as the pcRNA likewise failed to induce significant knockdown. Taken together, across all three cell types, long dsRNA and pcRNA consistently produced robust and statistically similar reductions in target transcript levels.

### Endocytosis inhibitors reveal differences in long dsRNA and pcRNA uptake

To assess whether long dsRNAs and pcRNAs rely on similar uptake routes, RNAi assays were performed in mosquito (CCL125), lepidopteran (Sf9), and fungal (*S. sclerotiorum*) cells under chemical inhibition of clathrin‑mediated endocytosis (CME). In all three systems, blocking CME with CPZ or BafA1 abolished knockdown by long dsRNA, leaving target transcript levels unchanged relative to controls (Fig 1). In contrast, pcRNAs retained substantial activity under CME inhibition, reducing target transcripts by ~50% in CCL125 cells, ~ 30% in Sf9 cells, and producing strong (>90%) knockdown in *S. sclerotiorum*. Disruption of clathrin‑independent pathways with methyl‑β‑cyclodextrin (MβCD) did not alter RNAi efficacy for either dsRNA type in any of the three species (S2 Fig). Together, these assays demonstrate that long dsRNA is strictly CME‑dependent, whereas pcRNA maintains RNAi activity through a CME‑independent uptake route.

### Visualization of dsRNA uptake in *A. aegypti, S. frugiperda* and *S. sclerotiorum*

To further assess the uptake and intracellular trafficking of long dsRNA and pcRNA, fluorescently labeled molecules were visualized in CCL125, Sf9, and *S. sclerotiorum* cells using confocal microscopy. In CCL125 cells, both long dsRNA and pcRNA were rapidly internalized, with diffuse cytoplasmic fluorescence evident within 20 min (Figs 2A and 2D, respectively). CPZ treatment markedly impaired long dsRNA entry, producing punctate fluorescence at or near the plasma membrane, consistent with disrupted CME (Fig 2B). BafA1 permitted uptake but retained long dsRNA within punctate vesicular structures, consistent with impaired progression into the cytoplasm. Although these vesicles are likely early endosomes, definitive identification would require co-localization with established endosomal markers. (Fig 2C). Cytoplasmic localization was quantified by comparing whole‑cell fluorescence with signal restricted to endosomal compartments using ImageJ. As expected, in the absence of clathrin inhibitors, more than 85% of labeled long dsRNA and pcRNA localized to the cytoplasm (Fig 2D and 2H). In contrast, only 30–38% of long dsRNA reached the cytoplasm when CME was inhibited. pcRNA, however, maintained strong cytoplasmic localization under both CPZ (Fig 2E and 2F) and BafA1 (Fig 2F and 2G), with more than 90% of fluorescence in the cytoplasm regardless of treatment (Fig 2H), demonstrating that its entry and trafficking are not hindered by inhibition of CME or endosomal acidification.

**Fig 2:**
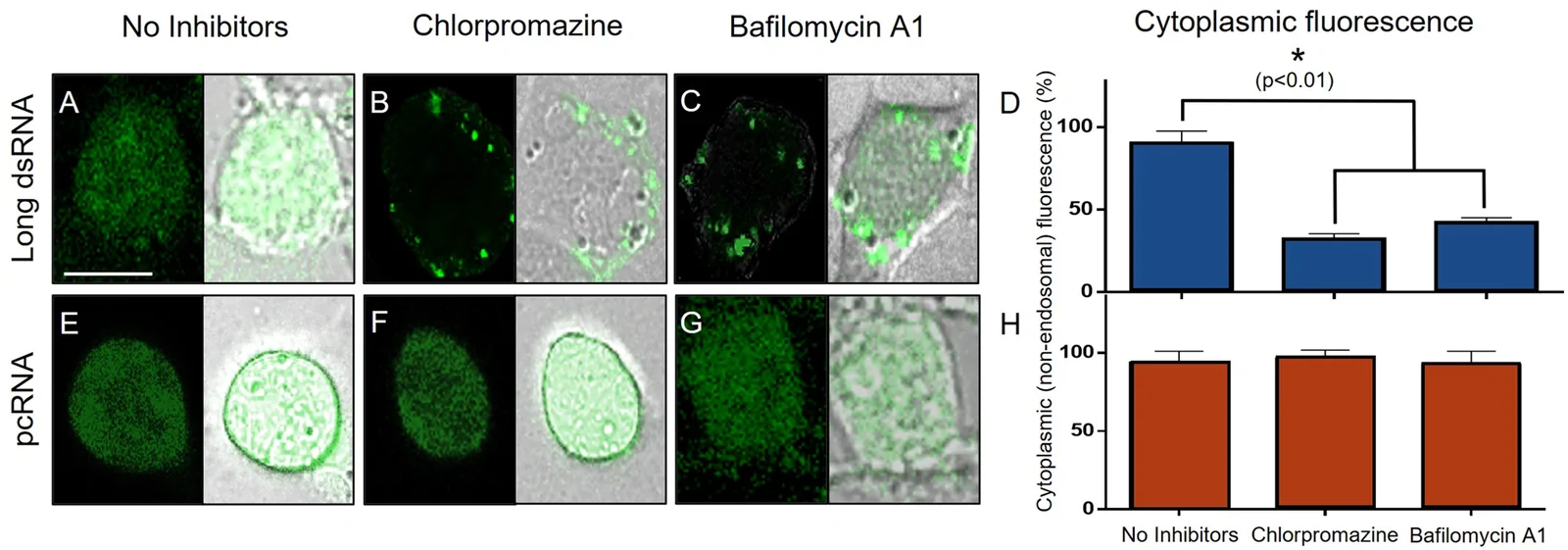
Cellular localization of fluorescein‑labelled long dsRNA and pcRNA in *Aedes aegypti* (CCL125) cells with and without using chemical inhibitors of clathrin-mediated endocytosis. Chemical inhibition of CME reduces uptake of exogenous long dsRNA, but not pcRNA, in *Aedes aegypti* (CCL125) cells. Confocal microscopy images show the intracellular localization of fluorescein-labelled long dsRNA (A-C) and pcRNA (E-G) under three conditions: (A, E) untreated controls; (B, F) chlorpromazine; and (C, G) bafilomycin A1. For each treatment, the left image in each panel shows the fluorescein channel alone, while the right image shows the merged fluorescein and brightfield channels. Images were acquired on a Zeiss LSM 700 confocal microscope with an Imager M2. Scale bar = 10 µm. (D and H) The percentage of total fluorescence is attributable to cytoplasmic signal, excluding the punctate endosomal aggregates. The values represent the means and SEM for 6 images, and the asterisk indicates significantly different values (One-way ANOVA, Tukey’s HSD, p < 0.05, that also show a large size effect, Hedges’ g > 2.0)..

A similar pattern was observed in Sf9 cells. Untreated cells showed rapid, diffuse cytoplasmic fluorescence for both long dsRNA and pcRNA (Figs 3A and 3E, respectively). CPZ again blocked long dsRNA internalization, restricting fluorescence to the cell periphery (Fig 3B), whereas BafA1 permitted uptake but trapped long dsRNA within vesicles (Fig 3C). pcRNA localization remained cytoplasmic under both inhibitor treatments (Figs 3F and 3G), indicating CME‑independent entry and avoidance of endosomal sequestration. Quantification of cytoplasmic fluorescence, measured by excluding signal concentrated in punctate, presumably endosomal aggregates, showed that 65–72% of long dsRNA and pcRNA localized to the cytoplasm in untreated cells. In contrast, only 23% (CPZ) or 35% (BafA1) of long dsRNA reached the cytoplasm under inhibitor treatments, with the remainder confined to punctate endosomal structures (Fig 3D). In contrast, pcRNA fluorescence in the cytoplasm remained steady at roughly 75%, unaffected by the presence or absence of either inhibitor (Fig 3H).

**Fig 3:**
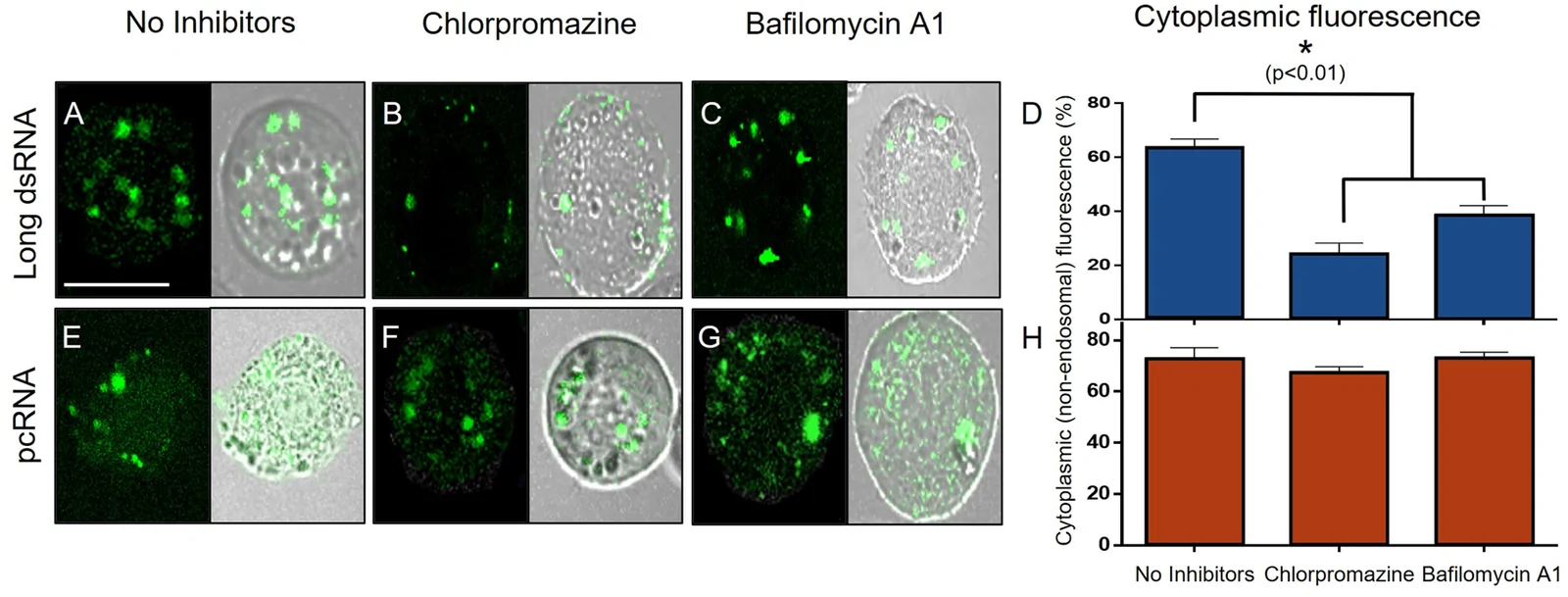
Cellular localization of fluorescein‑labelled dsRNA and pcRNAs in Spodoptera frugiperda (Sf9) cells with and without using chemical inhibitors of clathrin-mediated endocytosis. pcRNAs are internalized and reach the cytoplasm independent of CME, in contrast to the CME-dependent uptake of long dsRNA in *Spodoptera frugiperda* (Sf9) cells. Confocal microscopy images show the localization of fluorescein-labelled long dsRNA (A-C) and pcRNA (E-G) under three conditions: (A, E) untreated controls; (B, F) chlorpromazine; and (C, G) bafilomycin A1. For each treatment, the left image in each panel shows the fluorescein channel alone and the right image shows the merged fluorescein and brightfield channels. Images were acquired using a Zeiss LSM 700 confocal microscope with an Imager M2. Scale bar = 5 µm. (D and H) The percentage of total fluorescence is attributable to cytoplasmic signal, excluding the punctate endosomal aggregates. The values represent the means and SEM for 6 images, and the asterisk indicates significantly different values (ANOVA, Tukey post-hoc test, p < 0.05 that also show a large size effect, Hedges’ g > 2.0).

In *S. sclerotiorum*, fluorescein‑labeled long dsRNA and pcRNA were readily internalized into untreated hyphae, producing strong cytoplasmic fluorescence (Figs 4A and 4E, respectively). CPZ treatment prevented long dsRNA entry, restricting fluorescence to the hyphal periphery (Fig 4B), while BafA1 allowed uptake but retained long dsRNA within vesicles (Fig 4C). In contrast, pcRNA internalization was unaffected by CPZ or BafA1, with fluorescence distributed throughout the cytoplasm in all conditions (Fig 4F and 4G, respectively). As in the insect cells, quantification of cellular fluorescence confirmed that clathrin inhibition reduced cytoplasmic localization of long dsRNA, whereas pcRNA consistently remained cytoplasmic regardless of inhibitor treatment (Fig 4D and 4H)

**Fig 4:**
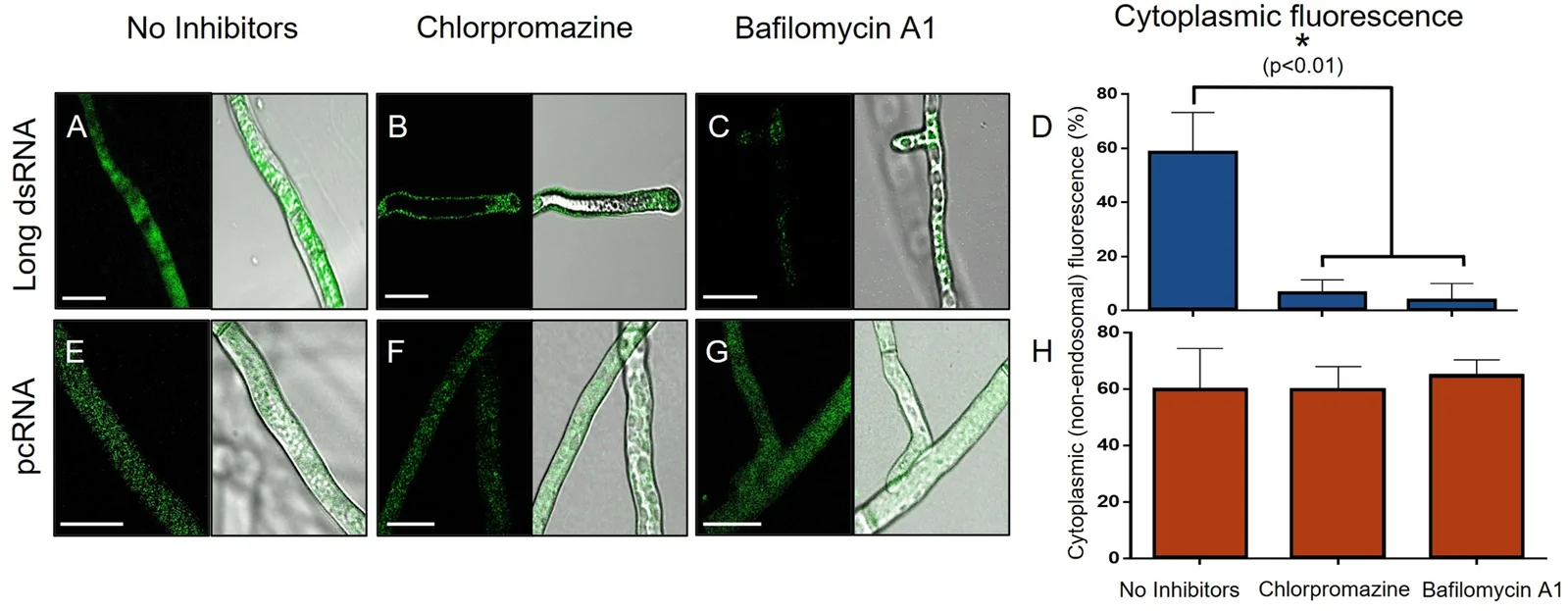
Cellular localization of fluorescein‑labelled long dsRNAs and pcRNAs in Sclerotinia sclerotiorum hyphae with and without using chemical inhibitors of clathrin-mediated endocytosis. CME is required for the uptake of long dsRNA, but not pcRNA, in *Sclerotinia sclerotiorum*. Confocal microscopy images show the localization of fluorescein-labelled long dsRNA (A-C) and pcRNA (E-G) in hyphae: (A, E) untreated controls; (B, F) chlorpromazine; and (C, G) bafilomycin A1. For each treatment, the left image in each panel shows the fluorescein channel alone and the right image shows the merged fluorescein and brightfield channels. Images were captured using a Zeiss LSM 700 confocal microscope with an Imager M2. Scale bars = 20 µm. (D and H) The percentage of total fluorescence attributable to cytoplasmic signal, excluding the punctate endosomal aggregates. The values represent the means and SEM for 6 images, and the asterisk indicates significantly different values (ANOVA, Tukey post-hoc test, p < 0.05 that also show a large size effect, Hedges’ g > 2.0)..

Treatment of the cells with MβCD, which disrupts lipid rafts and caveolae-mediated endocytosis, did not alter uptake of either dsRNA type in any of the three species S3 Fig.

These imaging results corroborate the functional assays: long dsRNA requires CME and subsequent endosomal escape to reach the cytoplasmic RNAi machinery, whereas pcRNA enters cells through a CME‑independent route that avoids endosomal trapping.

## Discussion

A critical factor that influences the efficiency of RNAi-based pest and pathogen control is the effective cellular uptake of dsRNAs and their subsequent release into the cytoplasm. Under optimal conditions, dsRNAs are released into the cytoplasm, where they initiate the RNAi pathway to suppress target gene expression. In this study, we combined cellular trafficking inhibition assays with confocal imaging to examine how conventional long dsRNAs and pcRNAs enter and localize within the cell. We complemented our findings with functional target gene knockdown experiments to confirm that the internalized molecules were successfully processed by the endogenous RNAi machinery. Collectively, these experiments allowed us to better understand and compare the uptake of long dsRNAs and pcRNAs across insect and fungal cell lines.

Our findings show that long dsRNAs are internalized primarily through CME in two significant agricultural pests, *Spodoptera frugiperda* and *Sclerotinia sclerotiorum* and a disease vector, *Aedes aegypti*. In these systems, the chemical inhibition of CME prevented the transport of long dsRNAs from the extracellular space into the cytoplasm as revealed by the marked reduction in fluorescent signals located in the cytoplasm of cells treated with CPZ or BafA1. We note that, without co‑localization using endosomal markers such as Rab5, Rab7, or LAMP‑1, we cannot definitively identify the specific vesicular compartments in which long dsRNA accumulated under inhibitor treatments. Nonetheless, long dsRNAs remained less accessible to the RNAi machinery resulting in the loss of RNAi-mediated target gene knockdown in distantly related yet economically important pests and pathogens. Our data provides visual evidence that a conserved cellular uptake mechanism involving clathrin is necessary for the intake of long dsRNA, which agrees with previous reports that arrived at the same conclusion [53–57].

Conversely, pcRNAs followed a markedly different uptake route. Prior work suggested that these compact structures enter cells through clathrin-independent mechanisms [49], and our results reinforce this conclusion across all three species examined. Despite pharmacological disruption of CME with CPZ or BafA1, pcRNAs continued to accumulate within the cytoplasm, exhibiting fluorescence patterns indistinguishable from untreated controls. This sustained internalization correlated with strong RNA activity; target transcripts were significantly reduced even under conditions that inhibited long dsRNA-mediated knockdown. Together, these findings indicate that pcRNAs bypass the clathrin-dependent pathway and access the cytoplasmic RNAi machinery through an alternative, CME-independent route, highlighting a mechanistic distinction that may contribute to their resilience across diverse cellular environments. Although species‑specific target genes were used, the inhibitor assays revealed a common pattern across all three systems: CME inhibition consistently blocked long dsRNA cellular distribution and knockdown, whereas pcRNA entry and silencing remained largely unaffected. This reproducible inhibitor response suggests that the cross‑kingdom differences in pcRNA efficacy are driven primarily by uptake‑route biology rather than by the identity of the target gene, though we acknowledge that gene‑specific factors may contribute to the magnitude of knockdown in individual species.

Endosomal escape represents an additional and well-documented barrier to effective RNAi in some insects [37,58]. Although long dsRNAs can be internalized efficiently across diverse taxa, their ability to exit endosomal compartments varies markedly. For example, lepidopteran cells such as *S. frugiperda* (Sf9) can exhibit substantially lower RNAi sensitivity than coleopteran cells, despite comparable uptake [58,59]. Shukla et al. [37] attributed this reduced responsiveness to the sequestration of exogenous long dsRNAs within endosomes, which prevents their processing into siRNAs and thereby blocks downstream gene silencing. Our results parallel this phenomenon. When endosomal acidification was inhibited using Baf-A1 in *S. sclerotiorum* and *A. aegypti* cells, long dsRNAs accumulated in vesicles and lost RNAi activity. These observations reinforce that productive RNAi depends on both efficient uptake and successful endosomal maturation, culminating in the release of long dsRNAs into the cytoplasm for engagement with the RNAi machinery.

We found that pcRNAs diverge sharply from the internalization behavior of long dsRNAs, as these compact molecules continued to reach the cytoplasm even when CME was inhibited in both insect and fungal systems. Their small, structured architecture could contribute to differences in how they interact with the cell surface, enabling access to alternative uptake routes or more rapid cytoplasmic entry. Notably, many RNAi‑sensitive invertebrates, including *D. virgifera*, *L. decemlineata* [60], and *C. elegans* [61], fail to internalize dsRNAs shorter than ~50 bp, which has contributed to the view that very small duplexes are generally ineffective mediators of systemic RNAi in insects. The pcRNAs used in this study, however, contain only a 23‑bp double‑stranded region, yet were readily internalized and remained fully competent for gene silencing. Interestingly, siRNAs of similar length did not induce knockdown unless delivered by transfection, despite being intact prior to treatment; this may reflect extracellular nuclease degradation or limited uptake of naked siRNA, possibilities consistent with prior reports of siRNA instability in insect and fungal systems. This contrast suggests that the unique secondary structure of pcRNAs may circumvent the lower size threshold that limits uptake of conventional short dsRNAs. At the opposite end of the spectrum, size‑dependent constraints have also been documented in filamentous fungi. In *Fusarium graminearum*, long dsRNAs (>1500 bp) exhibit markedly reduced fungicidal activity compared to shorter molecules (~400 bp) targeting the same gene, a difference attributed to poor cellular intake [62]. Together, these observations point to a size‑ and structure‑dependent landscape of dsRNA uptake and support the hypothesis that pcRNAs enter cells through clathrin‑independent pathways. However, the specific uptake pathway remains unresolved. Our inhibition assays indicate that caveolin‑ or lipid‑raft–mediated endocytosis, which can be disrupted by methyl‑β‑cyclodextrin, does not support internalization of either long dsRNA or pcRNA. Thus, alternative clathrin-independent pathways, such as macropinocytosis, micropinocytosis, scavenger‑receptor–mediated or other uptake mechanisms remain plausible candidates for pcRNA internalization. These possibilities may not be shared across insects and filamentous fungi, and defining the precise pathways in each system will require further investigation.

The ability of pcRNAs to retain full RNAi activity under conditions that impair long dsRNA uptake highlights their potential as a robust delivery platform for RNAi-based pest and pathogen control. In the systems examined here, the capacity of pcRNAs to bypass CME has important implications for resistance management. Resistance to RNAi-based insecticides may arise through reduced cellular uptake of exogenous dsRNAs rather than through mutations in the target gene. A notable example is the Colorado potato beetle (*L. decemlineata*), where a population developed >11,100-fold resistance to *V-ATPase subunit A* dsRNA within nine generations, accompanied by cross-resistance to dsRNAs targeting unrelated genes, which is consistent with a generalized defect in dsRNA internalization. Although no reported cases of dsRNA resistance have yet been reported in fungal pathogens, similar mechanisms are likely to emerge as RNAi-based fungicides become more widely deployed. The alternative uptake route used by pcRNAs directly addresses this vulnerability by circumventing the cellular bottlenecks that underline both natural RNAi recalcitrance and acquired resistance.

Taken together, our data indicate that pcRNAs enter insect and fungal cells through an uptake mechanism that remains to be elucidated but is clearly independent of CME. In addition to future studies aimed at resolving the uptake pathways used by different structured dsRNAs, it will be important to examine whether structural differences influence their durability within the cells, interaction with the RNAi machinery, and the persistence of siRNAs, as these factors may also shape their effectiveness in disrupting cellular function for pesticidal applications. Future work should also evaluate pcRNA performance in naturally recalcitrant fungal pathogens such as *Zymoseptoria tritici* and in resistant insect lines such as the aforementioned *Leptinotarsa decemlineata*. Such studies will be essential for determining whether SIGS approaches incorporating pcRNAs can introduce or restore RNAi sensitivity where conventional long dsRNA strategies fail. Overall, the strong gene silencing activity of pcRNAs, combined with their ability to access the cytoplasmic RNAi machinery through an alternative internalization route, positions this technology as a promising solution to both natural and acquired RNAi recalcitrance across diverse insect and fungal systems.

## Supporting information

S1 Fig Transcript knockdown by siRNA and pcRNA in cultured *Aedes aegypti* (CCL125) and *Spodoptera frugiperda* (Sf9) cells, and in *Sclerotinia sclerotiorum* hyphae, and the effect of lipofectamine on siRNA‑mediated knockdown in CCL125 and Sf9 cells. (A) pcRNAs, but not siRNAs, induce significant target gene knockdown in *Aedes aegypti* (CCL125), *Spodoptera frugiperda* (Sf9), and *Sclerotinia sclerotiorum*. Relative transcript abundance was measured for *Snf7* (*A. aegypti*), *PDZ* (*S. frugiperda*), and *AB-HYDROLASE 3* (*S. sclerotiorum*), normalized by ΔΔCq to *28S rRNA* (insects) or *Sac7* (fungus). Negative control (Neg) treatments consisted of cells or hyphae exposed to non‑specific (GUS) long dsRNA or pcRNA. Values show the means and standard errors from 5–6 biological replicates in insect cells, and 16 biological replicates in the fungus. Asterisks (*) indicate significant differences from the corresponding negative control (ANOVA with Tukey post-hoc test, p < 0.05). (B) Lipofectamine delivery of siRNAs confirms siRNAs can induce significant knockdown in insect cells when transfected; without transfection reagent, no significant knockdown was observed. Values represent the means and standard errors for 5–6 biological replicates. N denotes biological replicates for each treatment. Different letters denote significant differences (one-way ANOVA, Tukey HSD, p < 0.05 and a large size effect (Hedges’ g > 2.0).
(TIF)

S2 Fig Effects of lipid raft mediated endocytosis inhibition on long dsRNA and pcRNA induced gene knockdown in *Aedes aegypti* (CCL125), *Spodoptera frugiperda* (Sf9), and *Sclerotinia sclerotiorum.* CCL125 cells, Sf9 cells, and *S. sclerotiorum* hyphae were treated with long dsRNA or pcRNA in the presence of methyl-β-cyclodextrin (MβCD). (A) In CCL125 cells, MβCD treated dsRNA and pcRNA reduced *Snf7* transcript levels by 42% and 59%, respectively. (B) In Sf9 cells, long dsRNA and pcRNA reduced *PDZ* transcript levels by 34% and 59%, respectively. (C) In *S. sclerotiorum*, long dsRNA and pcRNA produced comparable knockdown of *AB HYDROLASE 3* (62% and 65%). Negative control (Neg) treatments consisted of cells or hyphae exposed to non‑specific (GUS) long dsRNA or pcRNA. Expression levels were quantified by ΔΔCq using *28S rRNA* (insects) or *Sac7* (fungus) as reference genes. N denotes biological replicates for each treatment. Different letters denote significant differences (one-way ANOVA, Tukey HSD, p < 0.05 and a large size effect (Hedges’ g > 2.0).
(TIF)

S3 Fig Confocal localization of fluorescein‑labelled long dsRNAs and pcRNAs in *Sclerotinia sclerotiorum* hyphae. Inhibition of lipid raft–mediated endocytosis does not affect uptake of long dsRNA and pcRNA in *Sclerotinia sclerotiorum*. Confocal images show intracellular localization of fluorescein labelled long dsRNA (A) and pcRNA (B) in hyphae treated with the lipid raft-disrupting inhibitor, methyl-β-cyclodextrin (MβCD). The left image in each panel shows the fluorescein channel alone and the right image shows the merged fluorescein and brightfield channels. Images were acquired on a Zeiss LSM 700 confocal microscope with an Imager M2. Scale bars = 20 µm.
(TIF)

S1 Table Primers and oligonucleotides used in generation of RNAs and qPCR analysis in *Sclerotinia sclerotiorum.* (DOCX)

S2 Table Primers and oligonucleotides used in generation of RNAs and qPCR analysis in *Aedes aegypti*, and *Spodoptera frugiperda.* (DOCX)
