# Supplementary material for: Paperclip RNAs bypass clathrin mediated endocytosis in insect and fungal cells
Source: PLoS One. 2026 Oct 28;21(10):e0359888. doi: 10.1371/journal.pone.0359888 (PMC13716168; doi:10.1371/journal.pone.0359888)
Supplement: Supplementary file 1 [file pone.0359888.s004.docx]

**S1 Table. Primers and oligonucleotides used in generation of alternative dsRNAs and qPCR analysis in *Sclerotinia sclerotiorum***

| ***AB-HYDROLASE 3* (*Sclerotinia sclerotiorum*)** NCBI accession XM_001597459.1 | |
| --- | --- |
| **Molecule** | **Sequence** |
| **Long dsRNA** | [T7]-TTCTGCCGGAAACCCTCTTC  [T7]-ACCGCCGATTGTGAAGACTT |
| **pcRNA** | [T7]-GTTCAAAAGCCTTAAGACTATCATCCAAAAAAAAAAAGGATGATAGTCTTAAGGCTTTTGAATGCATGAAAAAAAAACATG |
| **siRNA** | [T7]-GGATGATAGTCTTAAGGCTTTTGAA  [T7]-TTCAAAAGCCTTAAGACTATCATCC |
| **qRT-PCR** | CTTCCATCCCTGCCGCTTAC  CCATAGCGGCTCGATCTAGAATC |
| ***Sac7*** **(*Sclerotinia sclerotiorum*)** NCBI accession XM_001587270.1 | |
| **Molecule** | **Sequence** |
| **qRT-PCR** | CGATACTGTGCCTGTGACCA  CCTCTCCTCAAGCGCCATAG |
