# Supplementary material for: Paperclip RNAs bypass clathrin mediated endocytosis in insect and fungal cells
Source: PLoS One. 2026 Oct 28;21(10):e0359888. doi: 10.1371/journal.pone.0359888 (PMC13716168; doi:10.1371/journal.pone.0359888)
Supplement: Supplementary file 2 [file pone.0359888.s005.docx]

**S2 Table. Primers and oligonucleotides used in generation of alternative dsRNAs and qPCR analysis in *Aedes aegypti*, and *Spodoptera frugiperda***

| ***Snf7* (*Aedes aegypti*)** NCBI accession XM_021841452.1 | |
| --- | --- |
| **Molecule** | **Sequence** |
| **Long dsRNA** | [T7]-GAAATCTCCAACGCCGTCTC  [T7]-GATCATCATCATCCTCCGCAAC |
| **pcRNA** | [T7]-GCCTTGGCAGGCAGATCGGTTGCAAAAAAAAA GCAACCGATCTGCCTGCCAAGGCGACATGAAAAAAAAACATG |
| **siRNA** | [T7]-GCAACCGATCTGCCTGCCAAGGC  [T7]-GCCTTGGCAGGCAGATCGGTTGC |
| **qRT-PCR** | CGAAGTTGAATTGGACACTGC  GCTTCTTTCGTTTGAGTGCCT |
| ***28S rRNA* (*Aedes* *aegypti*)** NCBI accession XM_001648237.2 | |
| **Molecule** | **Sequence** |
| **qRT-PCR** | TCGCGACGTGGACATTATTC  GTGAAGGGTGCAGCTTACTT |
| ***PDZ* (*Spodoptera* *frugiperda*)** NCBI accession XM_035573157.2 | |
| **Molecule** | **Sequence** |
| **Long dsRNA** | [T7]-GCAAACGGAGTGGACAATAAC  [T7]-GTCCACCTAGTAGTTTCTTCATATC |
| **pcRNA** | [T7]-GCTATCTTTTGATAAAGTTCCTTGACAAAAAAAAA GTCAAGGAACTTTATCAAAAGATAGCAGCATGAAAAAAAAACATG |
| **siRNA** | [T7]-GTCAAGGAACTTTATCAAAAGATAGC  [T7]-GCTATCTTTTGATAAAGTTCCTTGACAT |
| **qRT-PCR** | AGACAATGGTGCTGGATATGCT  TTCTCGATGTGGTCTCCGAC |
| ***28S rRNA* (*Spodoptera* *frugiperda*)** NCBI accession XM_035581044.2 | |
| **Molecule** | **Sequence** |
| **qRT-PCR** | CTGCTTACAGAGACGAGGTTAAG  GGGTAATAGTCCAGACCAGAATTG |
